# Divergence and Convergence of Cerebral Ischemia Pathways Profile Deciphers Differential Pure Additive and Synergistic Mechanisms

**DOI:** 10.3389/fphar.2020.00080

**Published:** 2020-02-25

**Authors:** Penglu Wei, Pengqian Wang, Bing Li, Hao Gu, Jun Liu, Zhong Wang

**Affiliations:** ^1^ Institute of Basic Research in Clinical Medicine, China Academy of Chinese Medical Sciences, Beijing, China; ^2^ Institute of Chinese Materia Medica, China Academy of Chinese Medical Sciences, Beijing, China; ^3^ Institute of Information on Traditional Chinese Medicine, China Academy of Chinese Medical Sciences, Beijing, China

**Keywords:** additive effect, synergistic effect, signaling pathway, cerebral ischemia, pure mechanism

## Abstract

**Aim:**

The variable mechanisms on additive and synergistic effects of jasminoidin (JA)-Baicalin (BA) combination and JA-ursodeoxycholic acid (UA) combination in treating cerebral ischemia are not completely understood. In this study, we explored the differential pure mechanisms of additive and synergistic effects based on pathway analysis that excluded ineffective interference.

**Methods:**

The MCAO mice were divided into eight groups: sham, vehicle, BA, JA, UA, Concha Margaritifera (CM), BA-JA combination (BJ), and JA-UA combination (JU). The additive and synergistic effects of combination groups were identified by cerebral infarct volume calculation. The differentially expressed genes based on a microarray chip containing 16,463 oligoclones were uploaded to GeneGo MetaCore software for pathway analyses and function catalogue. The comparison of specific pathways and functions crosstalk between different groups were analyzed to reveal the underlying additive and synergistic pharmacological variations.

**Results:**

Additive BJ and synergistic JU were more effective than monotherapies of BA, JA, and UA, while CM was ineffective. Compared with monotherapies, 43 pathways and six functions were found uniquely in BJ group, with 33 pathways and three functions in JU group. We found six overlapping pathways and six overlapping functions between BJ and JU groups, which mainly involved central nervous system development. Thirty-seven specific pathways and 10 functions were activated by additive BJ, which were mainly related to cell adhesion and G-protein signaling; and 27 specific pathways and three functions of synergistic JU were associated with regulation of metabolism, DNA damage, and translation. The overlapping and distinct pathways and functions may contribute to different additive and synergistic effects.

**Conclusion:**

The divergence pathways of pure additive effect of BJ were mainly related to cell adhesion and G-protein signaling, while the pure synergistic mechanism of JU depended on metabolism, translation and DNA damage. Such a systematic analysis of pathways may provide an important paradigm to reveal the pharmacological mechanisms underlying drug combinations.

## Introduction

Stroke, one of the main causes of permanent disability and death worldwide, increased the total disease burden (Prabhakaran et al., 2015; Kim et al., 2015). As ischemic stroke is a complex disease with a series of mechanisms, multitarget drugs have been suggested to be more potent compared with single-target therapies (Yang et al., 2017; Wang et al., 2018). Therefore, combination therapy, as an effective pharmacological intervention, is still the trend, for example, the combination therapy with liposomal neuroprotectants and tissue plasminogen activator exerted a significantly higher neuroprotective effect compared with each treatment alone for the treatment of ischemic stroke (Fukuta et al., 2017). And many studies have shown that additive and synergistic drug combinations have greater advantages in improving efficacy, as well as reducing toxicity and adverse reaction in treating complex diseases, that is to say, the combination effects are larger than the sum of individual effects (Sato et al., 2010; Che et al., 2014; Al-Kuraishy et al., 2018; Bedenić et al., 2018). Exploring the additive and synergistic mechanisms of drug combination therapy is of great significance, by which we may understand the different tendencies of drug combinations, and design more appropriate combination regimens to achieve precision medicine (He et al., 2016).

Although several rigorous analysis methods for synergistic drug combinations have been proposed, such as chou-talalay combination index (CI) (Zhao et al., 2011), and isobolographic analysis (Jia et al., 2009), a single parameter–based research model may not clarify the molecular or systematic pharmacological mechanisms of combination therapies in a comprehensive fashion. Pathway analysis (PA) has possessed far-ranging applications in biomedical research, opening a new perspective of Omics data (Wadi et al., 2016). The PA approaches primarily seek to overcome the problem of interpreting lists of most important but isolated genes off the biological context that are the primary outputs of most basic high-throughput data analysis as differential expression analysis. This may provide a promising avenue for experimental high-throughput biological data to decipher combination therapies and subsequent hypothesis generation (García-Campos et al., 2015; Chen et al., 2016).

In previous studies, combination of BA and JA (BJ) have been demonstrated to exert an additive effect, while combination of JA and UA (JU) acted synergistically in treating cerebral ischemia (Chen et al., 2013; Yu et al., 2016; Zhang et al., 2016; Wang et al., 2018). The monocomponents of BA, JA, or UA was proved to have multiple pharmacological functions, such as antiinflammatory (Chen et al., 2018; Kang et al., 2018; Ko et al., 2019), antioxidant (Srinivas, 2010), neuroprotective (Wu et al., 2018; Li et al., 2018), antiplatelet (Lee et al., 2015), and protein response (Vang et al., 2014). Both of BA and JA regulated PI3K-Akt-PKB–BAD-CREB-PCREB pathway (Zhang et al., 2016); both of JA and UA acted on corticotropin releasing hormone signaling pathway (Chen et al., 2013). The combination therapy of JU exhibited both similarity and diversity in terms of signaling pathways. The additive effect of BJ was suggested to rest on the cross-talks of pathways from BA and JA at horizontal and vertical levels, including enhanced action in virus-mediated immune response (Wang et al., 2018). The synergistic effect of JU may regulate inflammation through a few key molecules in the network to treat ischemic stroke (Yu et al., 2016). All of these findings provide a hint for distinction between additive and synergistic effects. However, previous studies on combination therapies did not remove ineffective components, so the potential placebo effect could not be ruled out and the treatment outcomes of positive components might be interfered. In this paper, after excluding ineffective interference, we aim to further investigate the overlapping and diverse pharmacological mechanisms of BJ and JU at the signaling pathway level in treating cerebral ischemia. Based on GeneGo MetaCore analysis, we compared the pure pathways profiles of BJ and JU, as well as their cross-talks and functions, to systematically reveal the underlying pure mechanisms of additive and synergistic effects.

## Materials and Methods

### Animals and Middle Cerebral Artery Occlusion Model

Animal use protocols were reviewed and approved by the Ethics Review Committee for Animal Experimentation, China Academy of Chinese Medical Sciences, and the animal experiments were conducted in accordance with the Prevention of Cruelty to Animals Act 1986 and the National Institute of Health guidelines on the care and use of laboratory animals for experimental procedures. A total of 248 healthy adult male mice from Kunming, 3 months old, weighing 38–48 g, were used in this experiment. A focal cerebral ischemia-reperfusion model was induced after mice were anesthetized with 2% pentobarbital (4 mg/kg, intraperitoneally). The middle cerebral artery was occluded with an intraluminal filament for 1.5 h, followed by 24 h of reperfusion. The mice in the sham group proceeded with the same process, but the filament was not inserted. During the experiment, a heating pad was used to maintain the rectal temperature at 370°C–37.5°C. Brain temperature (monitored by 29-gauge thermocouples in the right corpus striatum) was maintained at 36°C–37°C under a temperature-regulating lamp; monitor the blood pressure, blood gas and glucose levels; EGGs were also monitored to ensure isoelectric currents during ischemia. Nine mice from each group were used to calculate the infarct ratio after 24-h reperfusion and the calculation of cerebral infarct volume was as described in our previous studies (Chen et al., 2012; Liu et al., 2012).

### Drug Administration

Animals were randomly divided into eight groups: Vehicle (0.9% NaCl), Sham (0.9% NaCl), CM (concha margaritifera; 50 mg/mL), BA (baicalin; 5 mg/ml), JA (jasminoidin; 25 mg/mL), UA (ursodeoxycholic acid, 7 mg/mL), BJ (a mixture of equal volumes of BA and JA), JU (a mixture of equal volumes of JA and UA). The herbal preparations were chemically standardized products provided by the China Natural Institute for the Control of Pharmaceutical and Biological Product or Beijing University of Traditional Chinese Medicine, and their composition was validated using fingerprint chromatographic methodologies. The herbal preparations were dissolved with 0.9% NaCl before use, and then injected into the tail vein 1.5 hs after Middle Cerebral Artery Occlusion (MCAO), at a dosage of 2 ml/kg based on body weight (Wang et al., 2011; Zhang et al., 2014).

### RNA Isolation and Microarray

The hippocampus of nine mice in each group was homogenized in TRIzol reagent (Invitrogen, USA). According to the single-step method, the total RNA was extracted (Chomczynski and Sacchi, 1987). RNA was further purified to remove genomic DNA contaminants and concentrated using the RNeasy Mini Kit (Qiagen, Valencia, CA, USA). RNA quality was assessed by measuring the 26 S/18 S ratio using a Bioanalyzer microchip (Agilent, Palo Alto, CA, USA). The collection procedures of 16,463 cDNA ischemia-related genes and the detailed process were described in our previous studies (Chen et al., 2013).

### Microarray Data Analysis

Experimental data was uploaded to the ArrayTrack system (U.S. Food and Drug Administration) for analysis through a robust multi-array analysis and standardization process. The results were submitted to the Array Express database. Genes with a *P* value less than 0.05 compared with CM group were identified for further analysis. Moreover, up-regulation or downregulation was indicated by the expression level of an increase >1.5-fold or a decrease <0.5-fold compared with CM group, respectively.

### Analysis of Pathways Profile

PA was conducted *via* MetaCore software (GeneGo Inc., division of Thomson Reuters) after the differentially expressed genes were identified. All differentially expressed genes were uploaded and mapped to the GeneGo database. *P* values were used to measure the significant genes and canonical pathways, which was calculated by Fisher's exact test. A lower p value indicated a higher correlation between the gene and the ontology category. The level of statistical significance was set at *P* < 0.05, which could screen out all the canonical pathways with a *P* < 0.05 and a fold change >1.5 for further analysis. We uploaded the list of significantly differentially expressed genes in the BA, JA, UA, BJ, and JU groups into the MetaCore software for functional PA. Pathway enrichment analyses were performed *via* the MetaCore software (defining an enriched pathway as having an enrichment *P* value < 0.05). All the enriched pathways were listed in **Supplementary Figure 1**. The function catalogue to which the pathways belong was defined according to the classification of the software itself. Pathways with identical functions were grouped into one category.

### Western Blotting

The hippocampus was removed from the brains of the nine mice in each group. Proteins (40 μg per lane) were separated by sodium dodecyl sulfate (SDS) polyacrylamide gel electrophoresis and transferred to nitrocellulose membranes (Hybond-C, Amersham, Buckinghamshire, UK) by electroblotting. Membranes were incubated in 5% nonfat milk for 1 h and incubated with antibodies to anti-*p53* (Santa Cruz), and developed using enhanced chemiluminescence (Amersham). The band density was measured by a GS-700 densitometer (Bio-Rad).

## Results

### Pharmacodynamics Effects of Reducing Ischemic Infarct Volume in Mice

Our prior experimental results indicated that infarction volume was significantly reduced after treatment with all compounds compared with the vehicle group except CM (*P* < 0.05, ANOVA). Thus, we considered the CM groups as a negative group, and the other groups as positive groups (Liu et al., 2012; Wang et al., 2015; Li et al., 2016). According the CI calculation, we found that JU exerted a synergistic pharmacological effect and BJ had an additive effect (Liu et al., 2012).

To explore the pure additive and synergistic mechanisms among the effective compounds by eliminating random interference from the ineffective drug, the differentially expressed genes used for further PA were obtained by comparing positive groups (including BA, JA, UA, BJ, and JU) with negative groups (CM).

### The Differential Pathways Profile of BJ and JU

#### Pure Additive Mechanism of BJ

**Figure 1:**
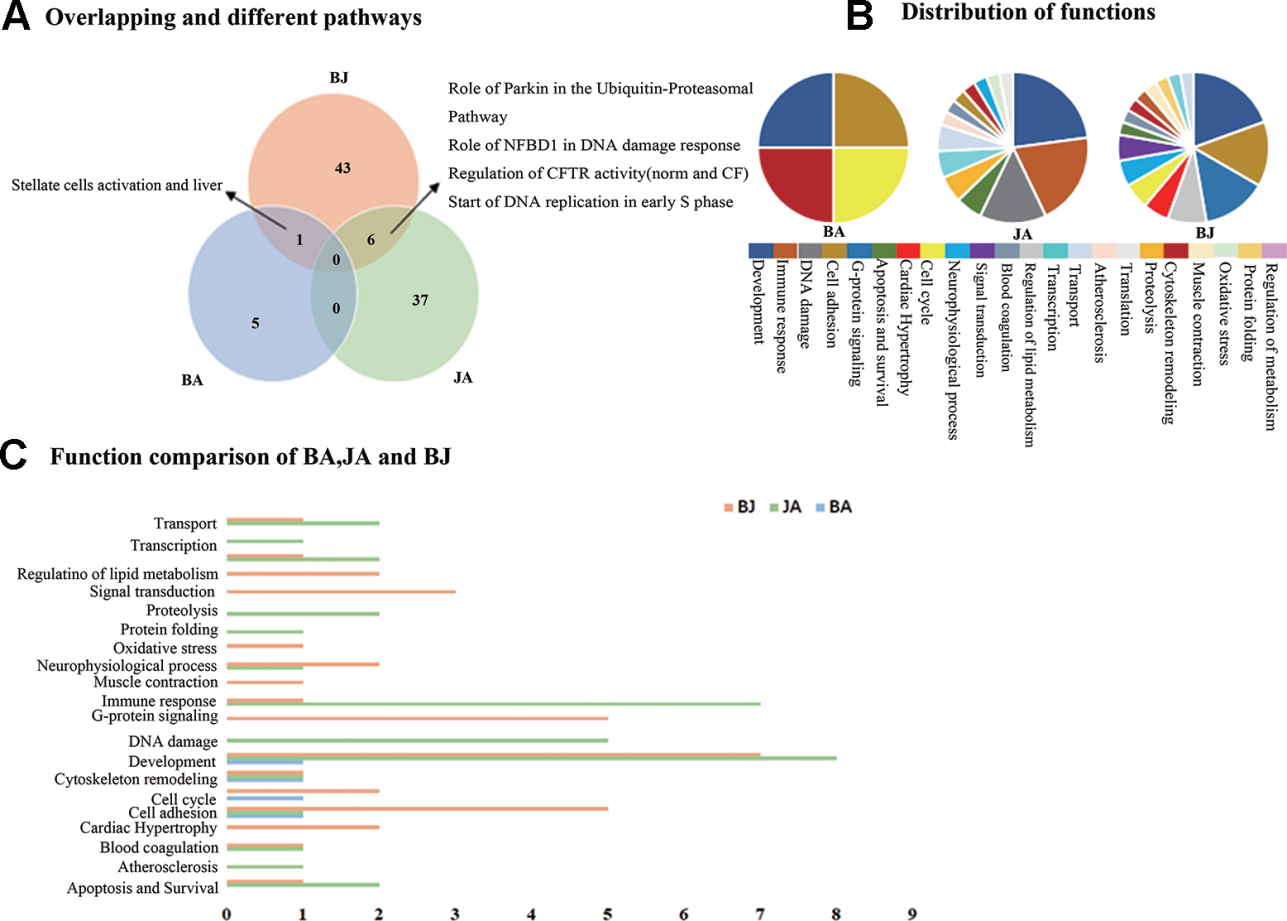
The Wayne figures of overlapping and different biological functions and pathways among groups. BA (blue) denotes BA vs. CM groups, JA (green) denotes JA vs. CM groups, and BJ (orange) denotes BJ vs. CM groups **(A)** The Wayne figures of overlapping and different pathways among BA, JA, and BJ. **(B)** The function distribution among BA, JA, and BJ. **(C)** The functional comparison among BA, JA, and BJ. Only significant functions are shown in this figure based on a P value <0.05 and a fold change =>1.5 between groups.

The combined effect produced by the action of two or more agents, being equal to the sum of their separate effects was referred to additive effect (Montes et al., 2011). In order to detect the generation of additive effect, we compared BJ with BA and JA (**Figure 1A**). We found 6, 43, and 50 significant pathways in the BA, JA, and BJ groups, respectively. In addition, there were one and six overlapping pathways between the BA and BJ, JA, and BJ groups, respectively, and no shared pathway existed between BA and JA or all the three groups. Compared with BA and JA, BJ produced 43.

Based on the functional annotations, we performed statistics on pathways with well-defined functional classification (**Figure 1B**). Thus, three molecular functional categories were shared among the three groups: cell adhesion, development and cytoskeleton remodeling. Additionally, there were one and six overlapping functions between BA and BJ, JA, and BJ groups, respectively. When BA was combined with JA, all of BA contributing functions (4) and some of JA contributing functions (6, 42.9% of the 14 functions in the JA group) were integrated, and another six functions emerged (**Figure 1C**): regulation of lipid metabolism, oxidative stress, G-protein signaling, cardiac hypertrophy, muscle contraction, and signal transduction. Interestingly, the overlapping functions in BJ with BA or JA (62.5% of all functions in the BJ group) cannot fully explain the functions found in the BJ group.

#### Pure Synergistic Mechanism of JU

**Figure 2:**
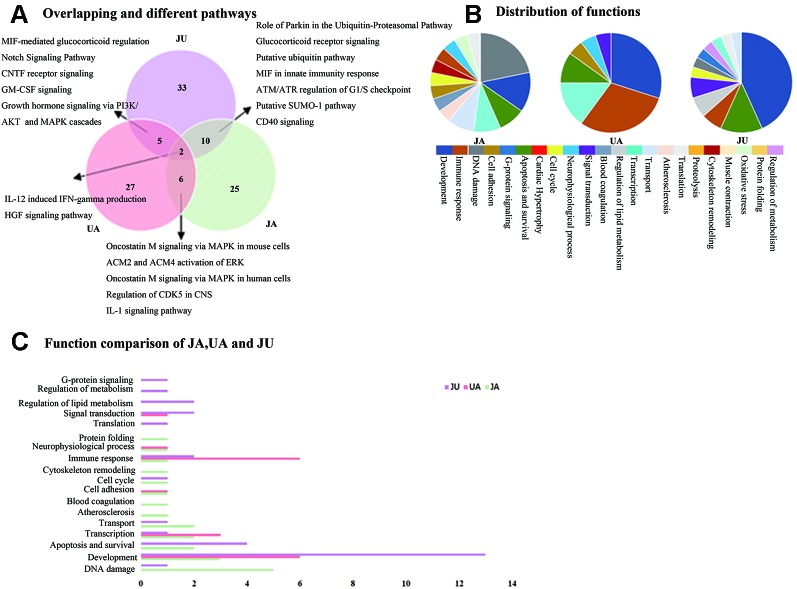
The Wayne figures of overlapping and different biological functions and pathways among groups. UA (red) denotes UA vs. CM groups and JU (purple) denotes JU vs. CM groups. **(A)** The Wayne figures of overlapping and different pathways among JA, UA, and JU. **(B)** The function distribution among JA, UA, and JU. **(C)** The functional comparison among JA, UA, and JU. Only significant functions are shown in this figure based on a P value <0.05 and a fold change >1.5 between groups.

Administration of combinations of drugs often produces unexpectedly large responses. This is known as synergy. It is often described as “1 + 1 > 2,” or supra-additive synergy (Geary, 2013). We compared the significant canonical pathways of the UA, JA and JU groups, respectively. Among these pathways in the three groups, we found 5, 10, and 6 overlapping significantly enriched pathways between JU and UA, JU and JA, and JA and UA groups, respectively; of which two pathways, i.e., IL-12-induced interferon-gamma (IFN-γ) production and hepatocyte growth factor (HGF) signaling pathway, were shared by all the three groups. Besides, 27, 25, and 33 specific pathways were noted in the UA, JA, and JU groups, respectively (**Figure 2A**).

The function distribution was listed in **Figure 2B**. Among the functional annotations of the three groups, there were 1, 4, and 2 overlapping functions between JU and UA, JU and JA, and JA and UA groups, respectively (**Figure 2C**). There were four overlapping functions (immune response, apoptosis and survival, transcription, and development) among the three groups. When UA was combined with JA, the majority of UA contributing functions (5, 71.4% of the seven functions in the UA group) and some of JA contributing functions (8, 57.1% of the 14 functions in the JA group) were integrated. Except for the overlapping functions, we also found some other functions (3, 25%) in JU group, including regulation of lipid metabolism, regulation of metabolism, and G-protein signaling.

### Variation of Pathways Profile Between BJ and JU

#### The Difference in Pathways and Functional Annotations

**Figure 3:**
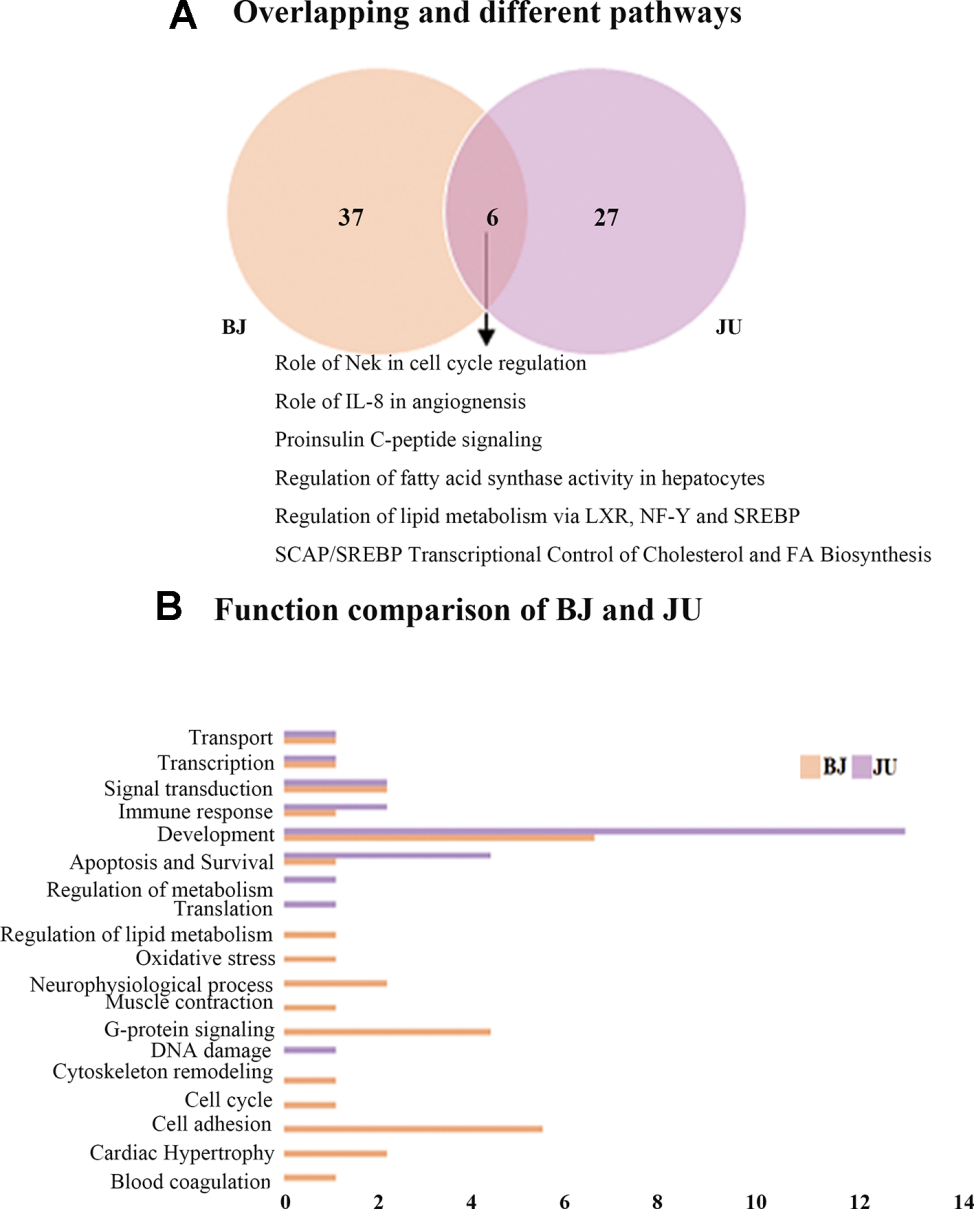
The Wayne figures of overlapping and different biological functions and pathways among groups. **(A)** The Wayne figures of overlapping and different pathways between BJ and JU. **(B)** The functional comparison between BJ and JU. Only significant functions are shown in this figure based on a P value <0.05 and a fold change >1.5 between groups.

There were 43 unique pathways in BJ group compared with BA or JA alone, and 33 unique pathways in JU group (**Figure 3A**). And then, we made a comparison between BJ and JU groups, and found six overlapping pathways as well as 37 and 27 nonoverlapping pathways between BJ and JU groups, respectively.

We compared the functions between BJ and JU groups, and discovered 16 and nine categories in BJ and JU groups, respectively (**Figure 3B**). A total of six functions were overlapping: development, signal transduction, immune response, apoptosis and survival, transport, and transcription. Interestingly, both of the first top function in BJ and JU groups was development, accounting for 6.25% and 11.1% of the total functions of BJ and JU groups, respectively. Moreover, there were 10 specific functions in BJ group, in which the top 2 categories were cell adhesion, and G-protein signaling, accounting for 12.5% of the total functions. The three distinctive functions in JU group were regulation of metabolism, translation, and DNA damage. These three categories made up a proportion of 33.3% of the total functions.

#### Variation of Targets in the Same Pathway

**Figure 4:**
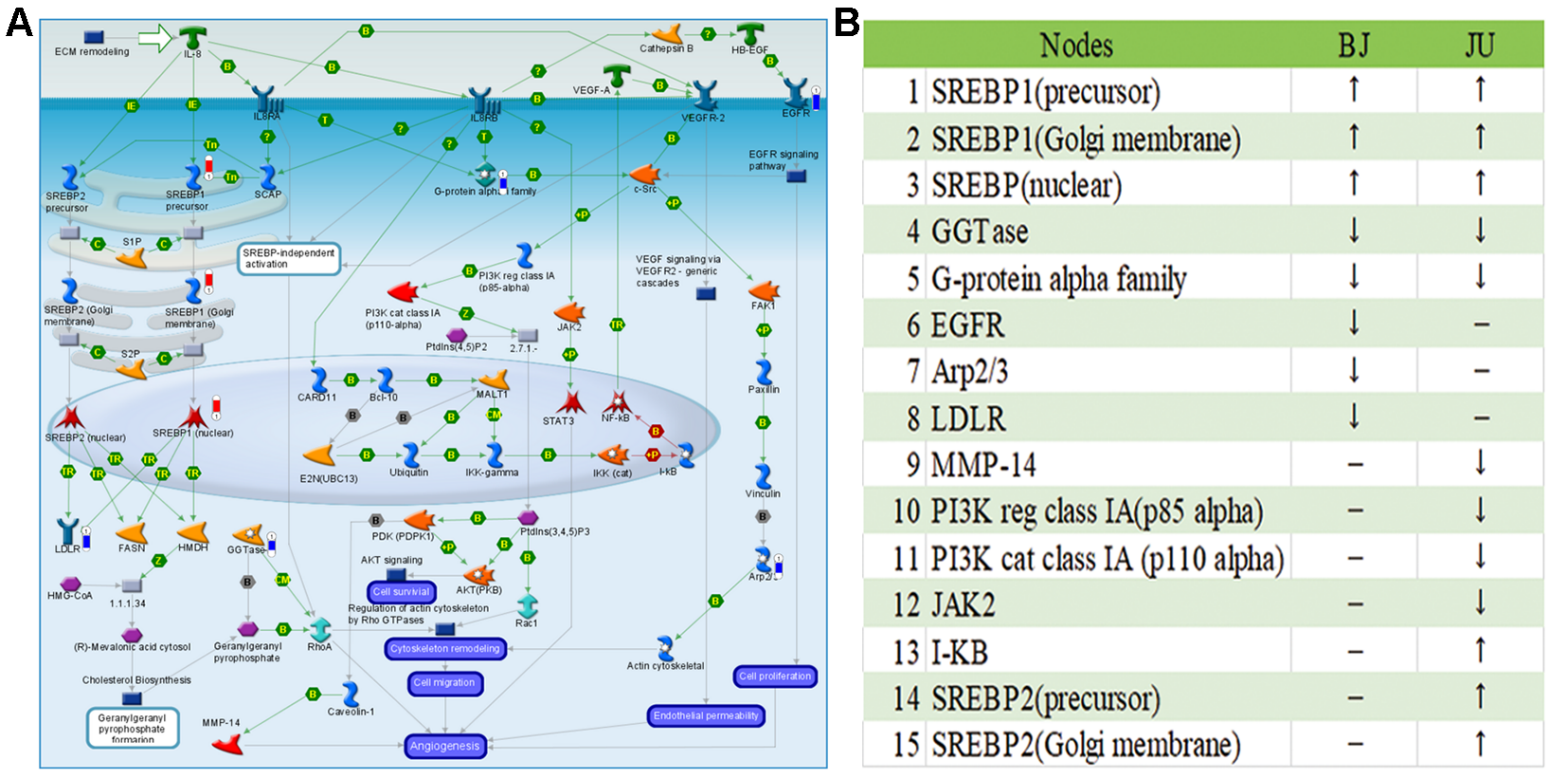
The “Development_Role of IL-8 in angiogenesis” pathway was shared by BJ and JU groups. **(A)** Differentially expressed genes regulating by BJ were labeled as thermometer-like figures, up-ward red thermometers indicate up-regulated signals, with height corresponding to significance level. The map legend can be viewed at http://pathwaymaps.com/pdf/ MC_legend.pdf. **(B)** The common and different targets regulated by BJ and JU group.

Although BJ and JU targeted on the same pathway, they showed diverse regulatory effects on this pathway. For example, we focused on the role of IL-8 in angiogenesis, which ranked first in JU group and ranked fourth in BJ group. In this pathway, there were 8 and 12 differentially expressed genes regulated by BJ and JU groups, respectively (**Figure 4A** and **Supplementary Figure 2**). Both BJ and JU acted on SREBP1 (precursor), SREBP1 (Golgi membrane), SREBP1 (nuclear), GGTase, and G-protein alpha family, in which SREBP1 (precursor), SREBP1 (Golgi membrane), and SREBP1 (nuclear) were upregulated, while GGTase and G-protein alpha family were down-regulated (**Figure 4B**). Besides these common molecules, BJ also regulated EGFR and LDLR, which were related to cell proliferation and dynamic balance of blood lipids, respectively (Cho et al., 2011); while JU regulated PI3K, JAK2, and I-κB, which were associated with cell immunity (Goodridge and Harnett, 2005).

#### The Different Vertical Converge of Pathways

**Figure 5:**
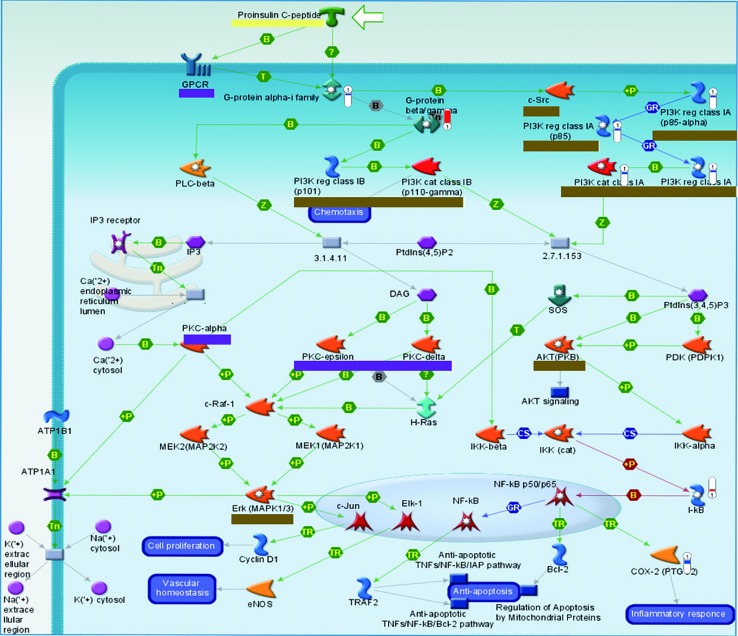
The vertical converge of pathways comparison. Pathways which are triggered by BJ and JU are labeled with purple and brown solid line box, respectively. Pathway triggered by both BJ and JU is labeled with yellow solid line box.

Proinsulin C-peptide signaling was the overlapping pathway between BJ and JU groups. In **Figure 5**, proinsulin C-peptide was a peptide segment of proinsulin which linked the carboxy terminus of the insulin B-chain to the amino terminus of the insulin A-chain (Wahren et al., 2000). C-peptide could initiate intracellular signaling cascades, which is usually related to the activation of a G protein-coupled receptor (GPCR), such as the activation of protein kinase (PKC) and the mobilization of calcium (Yosten et al., 2013). Extracellular signal regulated kinase (ERK) or phosphatidylinositol 3 kinase/protein kinase B (PI3K/AKT) pathways lied the downstream of the insulin receptor. In the pathway of AKT signaling only existing in JU group, PI3K was down-regulation and I-kB was up-regulation. However, in the pathway of ACM regulation of nerve impulse and Angiotensin activation of ERK *via* transactivation of EGFR only existing in BJ group, PKC was downregulation and Elk1 was upregulation. PI3K lied in the upstream of Proinsulin C-peptide signaling, while PKC and Elk in the downstream. The difference in targeting pathways vertically might contribute to the distinction between additive and synergistic effects in the treatment of cerebral ischemia.

### Various Regulation of P53 in Treatment Groups

**Figure 6:**
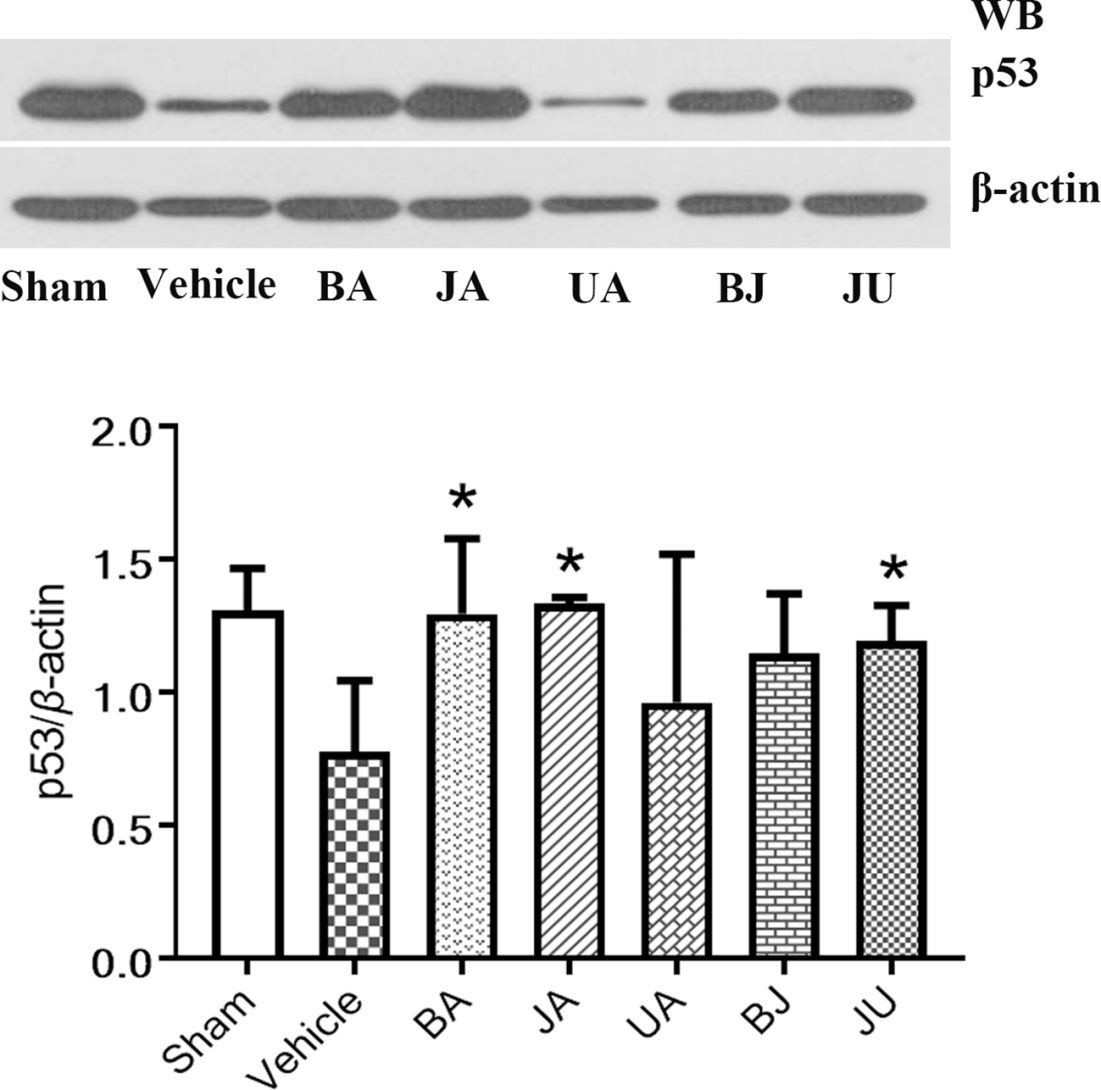
Active patterns of p53 under different treatment conditions. Western blot analysis was employed to identify the regulation of p53 among different treatment groups. Differences were determined using single-tail test. *P < 0.05 vs. vehicle.

p53 mRNA expression were altered in JA and JU groups, **Figure 6**. p53 was upregulated in JA and JU groups, while no significant changes in the UA group. The outcome was consistent with array-based gene expression measurements to confirm the reliability.

## Discussion

Our findings provide new insights into the mechanisms by which drugs influence multiple pathways in the ischemic area. We believe multitargets drugs were superior to single-target (Casci, 2004). Promising drug candidates should be combined to improve the efficacy of ischemic stroke treatment. In our research, a systematic analysis was performed to illustrate the additive and synergistic mechanisms underlying the combination therapies of BJ and JU in treating cerebral ischemia. We conducted comparative analyses based on GeneGo annotated pathways among the five treatment groups. Notably, overlapping and unique functions and pathways may elucidate the common and distinct mechanisms in different treatment groups.

### Variation of Pathway Vertical Converge Contributing to Diverse Mechanisms

Based on the cerebral ischemia target pathway network integrated from current knowledge, we found that the target pathways of drug combinations might form convergence rather than just stacking together. BJ activated the proinsulin C-peptide signaling and its upstream GPCR and PKC; while JU targeted this pathway and its downstream PI3K, AKT, and ERK. C-peptide, also known as a linker peptide, shares a common precursor with insulin-proinsulin. Several lines of evidence have suggested that the C-peptide receptor is a cell surface GPCR that is positively coupled to Ca^2+^ signaling (Grunberger et al., 2001). The neuroprotective effect of C-peptide was linked to mitogen-activated protein kinase (MAPK) pathways (Tam et al., 2006). Studies showed that insulin increased cell migration *via* ERK and PI3K/Akt pathways and influenced the vascular development (Wang et al., 2003); another research indicated that insulin and C-peptide made a difference to cell proliferation and apoptosis (Rizk et al., 2006), and attenuated inﬂammatory disorders of the vasculature (Mughal et al., 2010). The variation of pathway converge may attribute to difference between additive and synergistic effects, that is to say, “A signaling+B signaling” may act differently from “A signaling +C signaling” (**Figure 5**).

**Figure 7:**
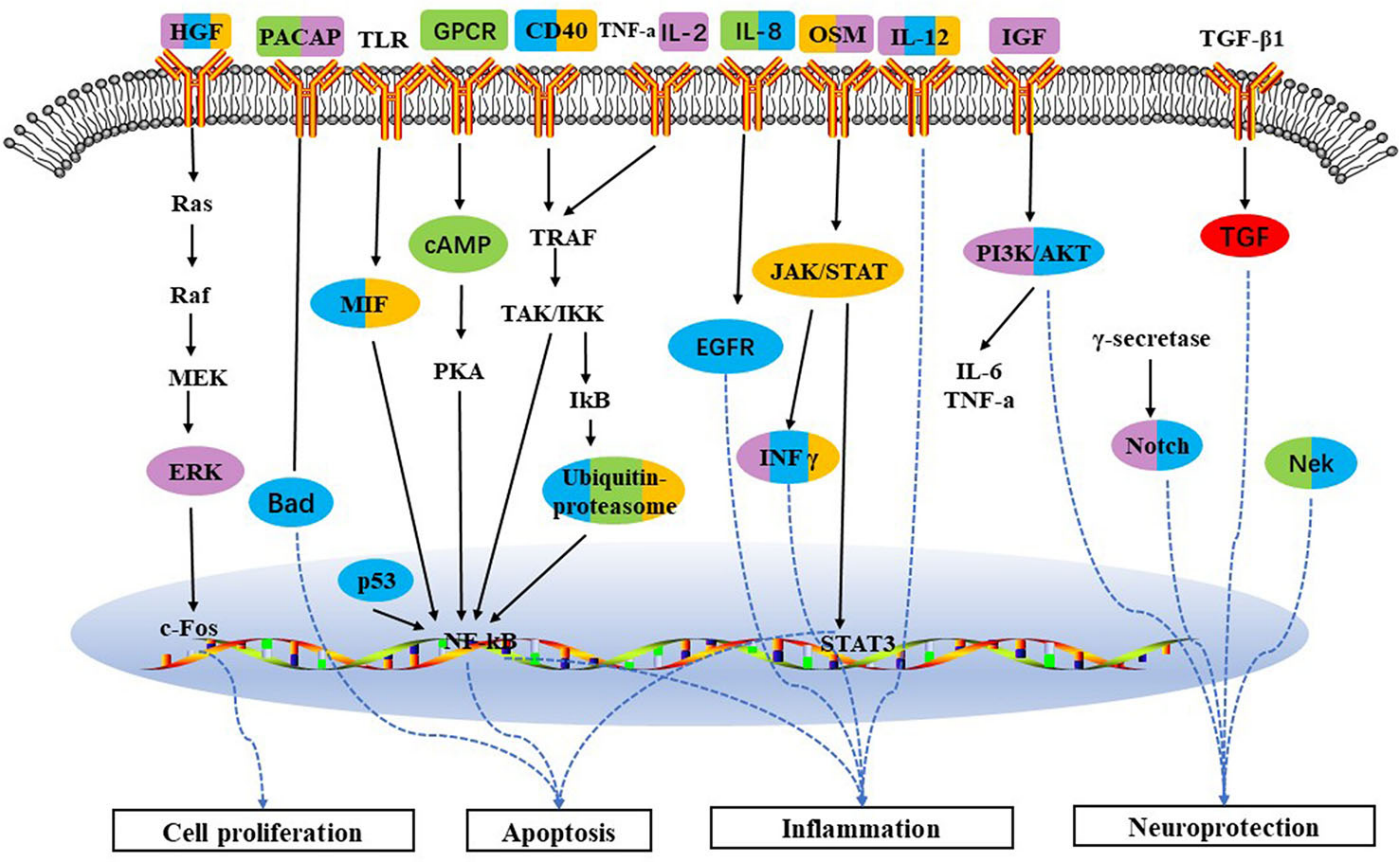
Schematic depicting target pathways identified for the five groups in treatment of cerebral ischemia. Pathways which are triggered by BA, JA, UA, BJ, and JU are labeled with red, orange, purple, green, and blue, respectively. Overlap of colors indicates the cotarget pathway for each group.

As for the pathway profiles, Role of Nek in cell cycle regulation and Role of Parkin in the Ubiquitin-Proteasomal Pathway were both identified in BJ and JU groups, which could partially explain the therapeutic effects of combination therapies. The ubiquitin proteasome system (UPS) plays a central role in the selective degradation of intracellular proteins. Among the key proteins regulated by the proteome include those involved in inflammatory process control, cell cycle regulation, and gene expression. There is now overwhelming data suggesting that UPS can cause cerebral ischemic damage (Wojcik and Di Napoli, 2004; Wang and Maldonado, 2006). And yet, IFN-gamma, MIF, CD40 were identified only in JU group. Similarly, PACAP was identified in BJ group. These functional pathways constituted the characteristic targeting profiles of BJ and JU, and jointly modulated inflammation, neuroprotection, apoptosis, and cell proliferation, with may lead to the diversity of additive and synergistic mechanisms. (**Figure 7**).

### The Common Functions of BJ and JU Concentrating on Central Nervous System Development

All the pathways of BJ and JU were primarily involved in development. For example, Endothelin-1(ET-1) (Moldes et al., 2012) and Dopamine D2 receptor(D2) (O'Neill et al., 1998) in BJ and GDNF (Glial cell line-Derived Neurotrophic Factor) in JU could generate neuroprotective effect. Reactive astrocytes in the ischemic hemisphere are involved in mechanisms that promote recovery and also express dopamine 1 (D1) and D2 receptors. An animal experiment shows that after stroke, the level of GDNF in the ischemic hemisphere of rats treated with levodopa increased, implicating that GDNF is involved in the mechanism of tissue reorganization and plasticity, and participates in the enhanced recovery of brain function loss by levodopa (Kuric et al., 2013), which provides overwhelming evidences that central nervous system development is of vital importance to ischemia stroke, so neuroprotective agent after stroke is considered a crucial pharmacological approach (Chechneva et al., 2014; de Oliveira et al., 2019).

Beyond that, signal transduction (Wei et al., 2018; Armstead et al., 2019), immunes response (Famakin, 2014), apoptosis and survival (Sun et al., 2018), transport (Li et al., 2018), and transcription, which existed in both BJ and JU groups, were also closely related to stroke. For example, during brain ischemia/reperfusion, intracellular signaling cascades were stimulated and interacted, which involved PI3K/AKT, Raf-1 and ERK1/2 (Zhou et al., 2015).

Interestingly, we noted that although BJ and JU modulated the same pathways, the targets of the two groups were not completely the same. For example, the IL-8 in angiogenesis signaling, BJ regulated molecules related to cell proliferation and dynamic balance of blood lipids; JU regulated molecules associated with cell immunity. This may partially explain the difference between additive and synergistic effects.

### The Differential Characteristic Functions of BJ and JU

As for characteristic mechanisms, the representative pathways only in BJ group were mostly related to cell adhesion and G-protein signaling, and those pathways in JU group were related to regulation of metabolism and DNA damage. For instance, triiodothyronine and thyroxine signaling were only enriched in the JU group; it has been shown that low free thyroxine index is associated with an increased risk for ischemic stroke and low free triiodothyronine levels are related to poor prognosis in acute ischemic stroke, which expressed the importance of metabolism regulation to stroke (Qureshi et al., 2006). Gap junctions just found in BJ group were related to cell adhesion; and it has been demonstrated in several studies that gap junction protein in astrocytes enhances the synaptic efficacy, which may promote functional recovery after stroke (Zhang et al., 2006).

In summary, after excluding the interference of the negative group, we obtained the pure mechanisms of BJ and JU combinations. Specifically, the difference between BJ and JU was embodied in three aspects: (i) diversity of targeting pathways: BJ mainly focused on cell adhesion and G-protein signaling; while JU mainly involved regulation of metabolism, DNA damage, and translation; (ii) activating different targets in the same pathway; (iii) variation of vertical converge of signaling pathways. Our method to systematic comparison of pathways for the pure mechanisms of different drug combinations may contribute substantially to the interpretation of the precise pharmacological actions of combination therapies.

## Data Availability Statement

The data generated in this article can be found in Arrayexpress, using the accession number E-TABM-6622.

## Ethics Statement

Animal use agreements were reviewed and authorized by the Ethics Review Committee for Animal Experimentation, China Academy of Chinese Medical Sciences, and the animal experiments were conducted in accordance with the Prevention of Cruelty to Animals Act 1986 and the National Institute of Health guidelines for conducting experimental procedures for the care and use of laboratory animals.

## Author Contributions

ZW and JL contributed to the conception or design of the work. PWe drafted the manuscript, and PWa with BL revised the manuscript. HG checked all aspects of this manuscript to ensure that we can contribute.

## Funding

The work was supported by the National Natural Science Foundation of China (Grant No. 81673833 and 81603401&81803966) and the Fundamental Research Funds for Central Public Welfare Research Institutes (Grant No. ZZ0908029, Z0469 and Z0547).

## Conflict of Interest

The authors declare that the research was conducted in the absence of any commercial or financial relationships that could be construed as a potential conflict of interest.

## Abbreviation

AKT, protein kinase B; BA, Baicalin; BJ, Baicalin combined with Jasminoidin; CM, Concha margaritifera; EGF, epidermal growth factor; EGFR, epidermal growth factor receptor; ERK, Extracellular signal regulated kinase; ET-1, Endothelin-1; GDNF, glial cell line-derived neurotrophic factor; GPCR, G protein-coupled receptor; HGF, production and hepatocyte growth factor; IFN-γ, interferon-gamma; IL, Interleukin; I-κB, Nuclear factor-κB inhibitor; JA, Jasminoidin; JAK, Janus kinase; JU, Jasminoidin combined with ursodeoxycholic acid; LDLR, low density lipoprotein receptor; MAPK, mitogen-activated protein kinase; MCAO, middle cerebral artery occlusion; MIF, macrophage migration inhibitory factor; PACAP, pituitary adenylate cyclase activating peptide; PKC, protein kinase; PI3K, phosphatidylinositol 3-kinase; SREBP, sterol regulatory element binding proteins; UA, Ursodeoxycholic acid; UPS, ubiquitin proteasome system.

## Supplementary Material

Supplementary Figure 1 The enriched pathways of BA vs CM, JA vs CM, UA vs CM, BJ vs CM, JU vs CM.

Supplementary Figure 2 The “Development_Role of IL-8 in angiogenesis” pathway was shared by BJ and JU groups. Differentially expressed genes regulating by JU were labeled as thermometer-like figures, up-ward red thermometers indicate up-regulated signals, with height corresponding to significance level. The map legend can be viewed at http://pathwaymaps.com/pdf/ MC_legend.pdf.
