# Supplementary figures and images for: Divergence and Convergence of Cerebral Ischemia Pathways Profile Deciphers Differential Pure Additive and Synergistic Mechanisms
Source: Front Pharmacol. 2020 Feb 25;11:80. doi: 10.3389/fphar.2020.00080 (PMC7053362; doi:10.3389/fphar.2020.00080)

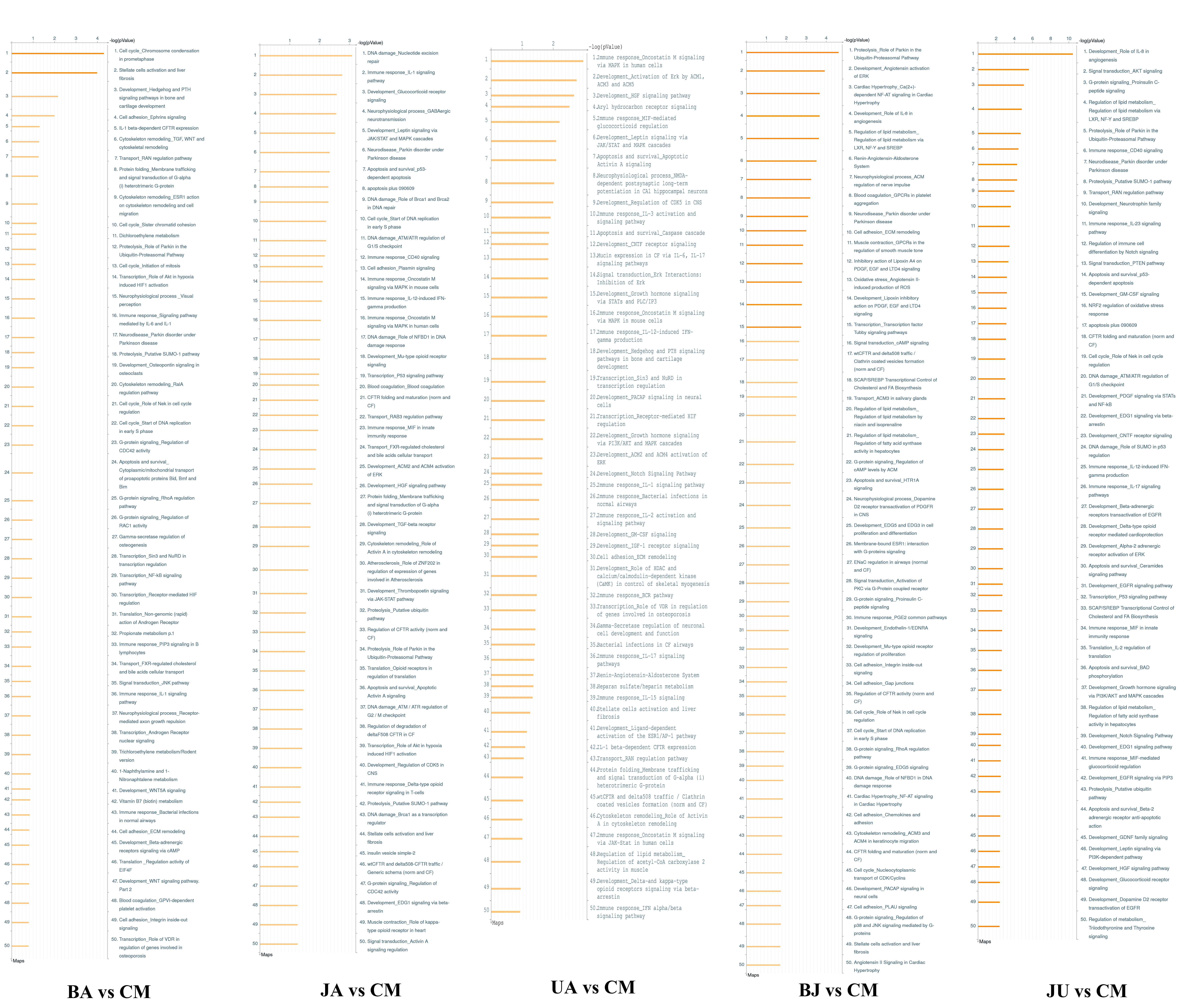

Supplement: Supplementary Figure 1 — The enriched pathways of BA vs CM, JA vs CM, UA vs CM, BJ vs CM, JU vs CM. [file Image_1.tif]
